# A Multiphysics Model for Predicting Microstructure Changes and Microhardness of Machined AerMet100 Steel

**DOI:** 10.3390/ma15134395

**Published:** 2022-06-21

**Authors:** Wenqian Zhang, Xupeng Chen, Chongwen Yang, Xuelin Wang, Yansong Zhang, Yongchun Li, Huan Xue, Zhong Zheng

**Affiliations:** 1 Hubei Key Laboratory of Modern Manufacturing Quality Engineering, School of Mechanical Engineering, Hubei University of Technology, Wuhan 430068, China; xp_chen@hbut.edu.cn (X.C.); 102010055@hbut.edu.cn (Y.Z.); a13091451416@163.com (Y.L.); stonemechanics@163.com (H.X.); zhengzh215@163.com (Z.Z.); 2 School of Mechanical Science and Engineering, Huazhong University of Science and Technology, Wuhan 430074, China; D202080301@hust.edu.cn (C.Y.); wangxl@hust.edu.cn (X.W.)

**Keywords:** surface integrity, microhardness, white layer, AerMet100 steel, machining

## Abstract

The machined-surface integrity plays a critical role in corrosion resistance and fatigue properties of ultra-high-strength steels. This work develops a multiphysics model for predicting the microstructure changes and microhardness of machined AerMet100 steel. The variations of stress, strain and temperature of the machined workpiece are evaluated by constructing a finite-element model of the orthogonal cutting process. Based on the multiphysics fields, the analytical models of phase transformation and dislocation density evolution are built up. The white layer is modeled according to the phase-transformation mechanism and the effects of stress and plastic strain on real phase-transformation temperature are taken into consideration. The microhardness changes are predicted by a model that accounts for both dislocation density and phase-transformation evolution processes. Experimental tests are carried out for model validation. The predicted results of cutting force, white-layer thickness and microhardness are in good agreement with the measured data. Additionally, from the proposed model, the correlation between the machined-surface characteristics and processing parameters is established.

## 1. Introduction

AerMet100 ultra-high-strength steel has the superior properties of high strength, good fatigue resistance and stress-corrosion resistance [1]. Hence, the steel is extensively used in manufacturing significant structural parts, such as carrier-aircraft landing gear [2]. The machined-surface integrities, including—but not limited to—microhardness, residual stress, surface roughness as well as microstructure changes, can seriously affect the service performance. For AerMet100 steel, the main microstructure changes are grain refinement, phase transformation and white-layer generation. The white layer appears featureless and white when observed in an optical microscope after standard metallographic preparation and has significantly high microhardness [3]. It has been widely considered that the microhardness and white layer have a greater impact on the machined surface and directly influence the fatigue strength and service life of the final products [4]. The main methods for studying the white layer and microhardness include experimental [5,6,7], finite-element [8,9,10] and analytical models [11,12].

The white-layer formation has been extensively studied. Zhang et al. [13] proposed that the white-layer formation was the result of a quick austenite transition and quenching process in hard cutting of AISI52100 steel. Meanwhile, the austenite transition of the white layer is enhanced by plastic deformation. Duan et al. [14] studied the white-layer formation mechanism through the experiments of orthogonal turning AISI52100 and AISI4340 steels. The results indicated that the white layer was generated due to the coupling effects of phase transition and plastic deformation. Wu et al. [15] found that the coupling thermal-mechanical effect during high-speed machining resulted in the generation of surface white-layer structure of hardened steels. According to the mechanism of white-layer formation, Arfaoui et al. [16] developed a FE model accounting for the phase-transformation mechanism and mechanical effects for evaluating the white-layer depth. Zhang et al. [17] established an orthogonal-cutting model of AISI52100 steel based on ABAQUS/EXPLICIT general software for predicting white-layer thickness. The phase-transition mechanism combined with the stress and strain was considered in the model. The mentioned prediction models about white-layer thickness were based on numerical simulation, which was time-consuming and dependent on computational resources.

As the result of phase transition and plastic deformation during machining, the microhardness change has been extensively researched, together with the microstructure changes. Ding et al. [18] employed the analytical method to predict the microhardness change according to the dislocation density model and dynamic phase-transformation process. Pan et al. [19] proposed a physics-based FE method to model the phase change as well as the increase in grain size induced by the machining of Ti-6Al-4V alloy. Bouissa et al. [20] established a microstructure-based 3D FE model for predicting the microhardness distribution and phase-volume fractions of high-strength steel forgings during the water-quenching process. These mentioned FE models cannot predict the microhardness change directly. The analytical models are very complicated for the evaluation of the stress, strain and temperature fields.

To improve resultant surface integrity by effectively selecting the machining process parameters, it is necessary to develop a model for evaluating the surface characteristics and correlating them to the processing parameters. Therefore, this study aims to provide a multiphysics model for predicting the white layer and microhardness by relating the phase transformation and dislocation density evolution to the cutting parameters and workpiece material properties. Through developing an analytical framework based on the relationship between the thermomechanical load and the white-layer generation as well as the microhardness change, the white-layer thickness and microhardness are predicted by applying the stress, strain and temperature fields that are evaluated from FE analysis. Finally, the proposed models are validated through a detailed experimental investigation on AerMet100 steel.

## 2. Multiphysics Modeling of Machining

### 2.1. Analytical Models for White Layer and Microhardness

Accounting for the combined effects of plastic deformation and phase transition on material evolution, analytical models for white-layer generation and microhardness changes are established based on the variations of stress, strain and temperature during machining.

#### 2.1.1. Model of White-Layer Generation

**Figure 1:**
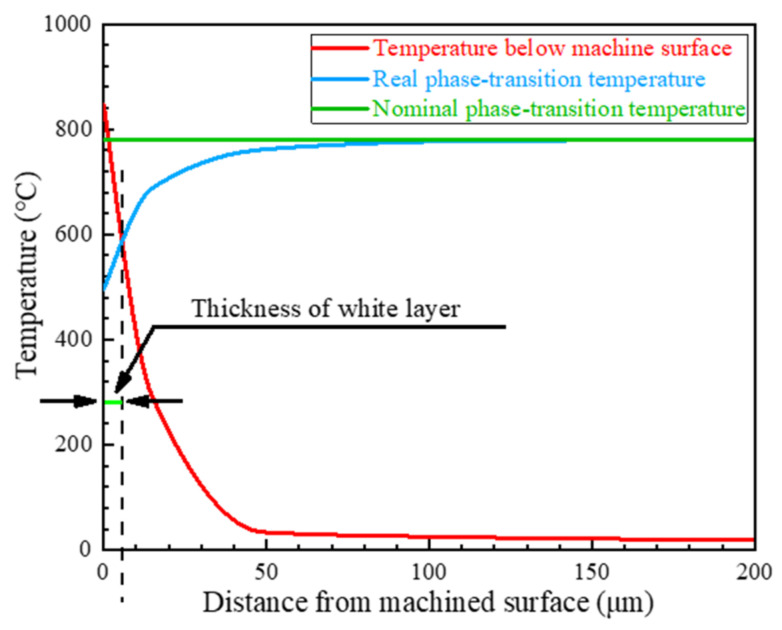
Prediction model of the white-layer thickness.

As proposed by Zhang et al. [13], the white-layer generation is the result of a rapid austenite transformation and quenching process, which is influenced by plastic deformation. Based on this mechanism, the white-layer thickness can be evaluated by combining the workpiece temperature distribution with the real transformation-temperature variation, which is related to the stress and strain state, as illustrated in Figure 1.

Based on the study reported by Zeng et al. [21], the real phase-transition temperature at point M can be expressed by,

(1) $$T\left(x,y\right)=T_{0}\exp\left(\frac{\sigma_{eq}\left(x,y\right)\Delta_{\alpha ’}^{\gamma }V-W\left(x,y\right)}{\Delta_{\alpha ’}^{\gamma }H}\right)$$

where $\Delta_{\alpha^{\prime }}^{\gamma }V$ is molar volume increment and $\Delta_{\alpha^{\prime }}^{\gamma }H$ is molar latent heat during α′→γ transformation. *T*_0_ is the nominal transformation temperature of austenite, which is about 780 °C for AerMet100 steel. $\sigma_{eq}$ is reported according to von Mises equivalent stress equation [22], which can be expressed as below:

(2) $$\sigma_{eq}=\frac{1}{\sqrt{2}}\sqrt{{\left(\sigma_{x}-\sigma_{y}\right)}^{2}+{\left(\sigma_{y}-\sigma_{z}\right)}^{2}+{\left(\sigma_{z}-\sigma_{x}\right)}^{2}+6\sigma_{xy}{}^{2}}$$

According to Duan et al. [22], $\Delta_{\alpha^{\prime }}^{\gamma }V$ is −0.06 cm^3^/mol and the $\Delta_{\alpha^{\prime }}^{\gamma }H$ of pure iron, which equals 920.5 J/mol, is used, as the α′→γ transition of AerMet100 steel is similar to that of pure iron. For pure iron, the units of strain-energy density are unified by the formula 1 J(m^3^)^−1^ = 7.377 × 10^−6^ J(mol)^−1^. *W* is the strain-energy density at point M, which plays a significant role in the real transformation-temperature. Based on elastoplastic mechanics, the plastic strain-energy density is obtained by

(3) $$W^{p}\left(x,y\right)=\sum_{x_{i}=x_{s}}^{x}\sigma_{eq}(x_{i},y)d\varepsilon_{eq}^{p}$$

where $d\varepsilon_{eq}^{p}$ represents the equivalent plastic strain increment. Combined with the above relationships, the real phase-transformation temperature during machining under specific stress and strain can be described as below:

(4) $$T\left(x,y\right)=780\exp\left(\frac{-0.06\times 10^{-6}\sigma_{eq}\left(x,y\right)-7.377\times 10^{-6}W^{p}\left(x,y\right)}{920.5}\right)$$

Therefore, a function $\Delta t_{wl}$ is defined to determine the value of white-layer thickness. It is assumed that the white-layer thickness is equal to the maximum depth where phase transformation occurs.

(5) $$\Delta t_{wl}\left(x\right)=\max\left[T_{w}\left(x,y\right)-T\left(x,y\right)\geq 0\right]\left(0\leq y\leq 10\right)$$

where *y* is the maximum *y* coordinate of researched points. Considering that the white-layer thickness is typically a few microns, *y* is set within 10 µm in this study.

#### 2.1.2. Model of Microhardness Change

According to Ding et al. [18], both the severe plastic deformation and the dynamic phase transition could be responsible for the microhardness variation during cutting. Therefore, the resultant microhardness change Δh can be expressed by the sum of microhardness changes induced by severe plastic deformation ($\Delta h_{SPD}$) and dynamic phase transformation ($\Delta h_{DPT}$):

(6) $$\Delta h=\Delta h_{SPD}+\Delta h_{DPT}$$

Determined by the dislocation density, the strengthening of material microhardness resulted from severe plastic deformation is expressed as

(7) $$\Delta h_{SPD}=k_{h}M_{t}\alpha_{h}Gb\sqrt{\rho_{tot}}$$

where $k_{h}$ is a constant slope of 0.5 and $\alpha_{h}$ is a constant of 0.25. Parameters *M_t_*, *G* and *b* are the Taylor factor, shear modulus and magnitude of the Burgers vector of the material, respectively.$\rho_{tot}$ is the total dislocation density, which is determined by both cell interiors and walls densities,

(8) $$\rho_{tot}=f_{w}\rho_{w}+\left(1-f_{w}\right)\rho_{c}$$

Based on the dislocation-density model [18], a dislocation cell structure is assumed to form during plastic deformation. The evolution rates of dislocation density in cell interiors (${\dot{\rho }}_{c}$) and cell walls (${\dot{\rho }}_{w}$) can be expressed by

(9) $$\begin{array}{l} {\dot{\rho }}_{c}=\frac{\alpha^{*}{\dot{\gamma }}_{w}^{r}\sqrt{\rho_{w}}}{\sqrt{3}b}-\frac{6\beta^{*}{\dot{\gamma }}_{c}^{r}}{db{\left(1-f_{w}\right)}^{\frac{1}{3}}}-k_{0}{\dot{\gamma }}_{c}^{r}\rho_{c}{\left(\frac{{\dot{\gamma }}_{c}^{r}}{{\dot{\gamma }}_{0}}\right)}^{-\frac{1}{n_{0}}}\\ {\dot{\rho }}_{w}=\frac{6\beta^{*}{\dot{\gamma }}_{c}^{r}{\left(1-f_{w}\right)}^{\frac{2}{3}}}{dbf_{w}}+\frac{\beta^{*}{\dot{\gamma }}_{c}^{r}\left(1-f_{w}\right)\sqrt{3\rho_{w}}}{bf_{w}}-k_{0}{\dot{\gamma }}_{w}^{r}\rho_{w}{\left(\frac{{\dot{\gamma }}_{w}^{r}}{{\dot{\gamma }}_{0}}\right)}^{-\frac{1}{n_{0}}} \end{array}$$

where $\alpha^{*},\beta^{*}$ and $k_{0}$ are control parameters of dislocation evolution rate, *n*_0_ is a temperature-sensitivity parameter and *d* is the dislocation cell size. ${\dot{\gamma }}_{c}^{r}$, ${\dot{\gamma }}_{w}^{r}$ and ${\dot{\gamma }}_{0}$ are the resolved shear-strain rates for cell interiors, resolved shear-strain rates for cell walls and the reference resolved shear-strain rate, which are assumed to follow the equation: ${\dot{\gamma }}_{c}^{r}={\dot{\gamma }}_{w}^{r}={\dot{\gamma }}^{r}$. The ${\dot{\gamma }}^{r}$ is related to von Mises strain rate as ${\dot{\gamma }}^{r}=M_{t}\dot{\varepsilon }$. $f_{w}$ is the volume fraction of the dislocation cell wall and determined by

(10) $$f_{w}=f_{\infty }+\left(f_{0}-f_{\infty }\right)e^{-\frac{\gamma^{r}}{{\tilde{\gamma }}^{r}}}$$

where $f_{0}$ and $f_{\infty }$ are defined as the initial and saturation volume fractions of cell walls, respectively. ${\tilde{\gamma }}^{r}$ represents the reduction rate of $f_{w}$. The average cell size *d* is relevant to total dislocation density,

(11) $$d=\frac{K_{0}}{\rho_{tot}}$$

where *K*_0_ is a material constant.

In addition, as proposed by Zhang et al. [23], the microhardness change involved by dynamic phase transformation can be estimated by

(12) $$\Delta h_{DPT}=\sum f_{i}h_{i}-h_{0}$$

where $f_{i}$ and $h_{i}$ represent the phase fraction and microhardness of phase i. $h_{0}$ is the initial matrix microhardness. For AerMet100 steel, the subsurface phase consists of austenite (γ) and martensite ($\alpha^{\prime }$). Therefore, the microhardness variation is determined by

(13) $$\Delta h_{DPT}=f_{\alpha^{\prime }}\left(h_{\alpha^{\prime }}-h_{\gamma }\right)$$

During cooling, the following equation [18] is employed to evaluate the martensite fraction when the workpiece temperature drops below the temperature of martensite formation:

(14) $$f_{m}=f_{\gamma }^{*}\{1-\exp[-0.011\left(M_{s}-T_{w}\right)]\}$$

where $f_{m}$ is the martensite volume fraction at the current temperature $T_{w}$. $f_{\gamma }^{*}$ represents the austenite volume fraction when the workpiece temperature reaches the onset temperature of martensitic transformation $M_{s}$. It is essential to note that this model is based on the assumption of a complete austenitizing when the workpiece temperature exceeds the real transformation temperature. The differential of the above equation in the form of increments [24] can be used in the present study for tracing the martensite volume fraction numerically during cooling,

(15) $$\Delta f_{m}=\left\{-0.011\exp\left[0.011(T_{w}-M_{s})\right]\right\}\Delta T_{w}\left(T_{w}\leq M_{s}\right)$$

in which $\Delta T_{w}$ is the temperature increment.

It should be pointed out that when the workpiece temperature does not reach the real phase-transition temperature, tempering under different temperature gradients would also cause a significant change in the material microhardness [25]. Therefore, when evaluating the microhardness in the tempering zone, the phase transitions due to tempering need to be considered. According to the chemical compositions of AerMet100 steel, the microhardness change in the tempering zone can be estimated by the relationship proposed by Grange et al. [25],

(16) $$\Delta h_{DPT}^{*}=-0.66*T_{w}+392-h_{0}\left(149\leq T_{w}<T\right)$$

### 2.2. Finite-Element Model for Orthogonal Cutting

**Figure 2:**
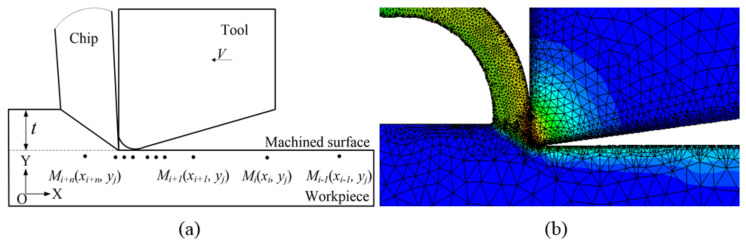
The model diagrams. (**a**) Schematic diagram of orthogonal cutting; (**b**) the deformed mesh configuration.

To obtain the histories of stress, strain and temperature variations during machining, a FE model for orthogonal cutting of AerMet100 steel is built up, as shown in Figure 2. It is noted that, at any depth, the stress, strain and temperature at point *M_i+1_* (*x_i+1_*, *y_j_*) are equivalent to those at point *M_i_* (*x_i_*, *y_j_*) after time Δ*t*, as shown in Figure 2a. Based on this relationship, the histories of stress, strain and temperature changes at any depth can be obtained by FE analysis. The workpiece and tool are meshed with triangular continuum elements in the FE model, as shown in Figure 2b.

As reported by Li et al. [26], the Johnson–Cook model is used to describe the material constitutive relationship, which defines the flow stress as a function of equivalent strain, strain rate and temperature as follows:

(17) $$\bar{\sigma }=\left[A_{jc}+B_{jc}{\left(\bar{\varepsilon }\right)}^{n_{jc}}\right]\left[1+C_{jc}\ln\left(\frac{\overset{\cdot }{\bar{\varepsilon }}}{\overset{\cdot }{\bar{\varepsilon_{0}}}}\right)\right]\left[1-{\left(\frac{T_{w}-T_{r}}{T_{m}-T_{r}}\right)}^{m_{jc}}\right]$$

where *A_jc_*, *B_jc_*, *C_jc_*, *m_jc_* and *n_jc_* are the J-C model parameters. *T_m_*, *T_r_* and *T_w_* represent the melting temperature of the workpiece material, room temperature and workpiece temperature, respectively.

In addition, a simple friction model based on the constant Coulomb friction law [27] is implemented in the FE code. The friction coefficient μ equals 0.3 in the present FE model, which is based on the satisfactory results between predicted and experimental cutting forces [28]. The FE numerical procedure is performed under the assumptions: rigid cutting tool and isotropic material.

**Figure 3:**
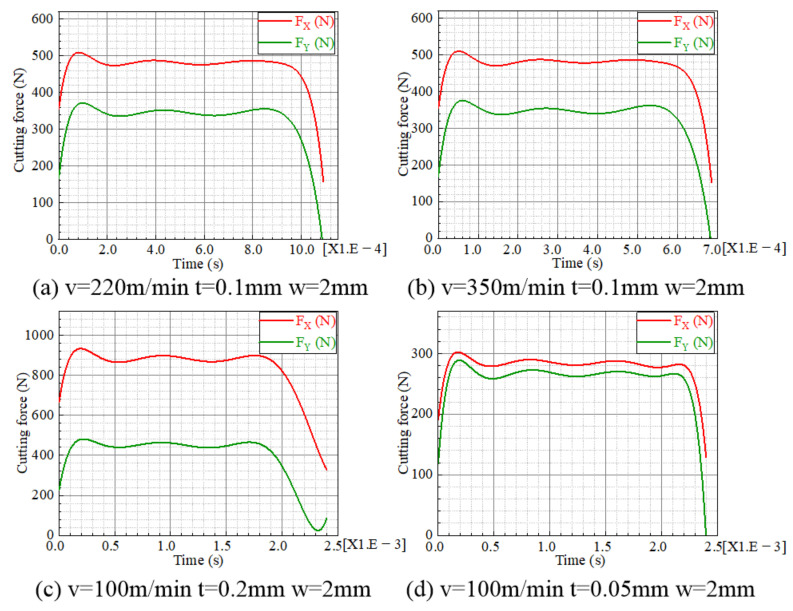
Cutting-force curves. (**a**) *V* = 220 m/min *t* = 0.1 mm *w* = 2 mm, (**b**) *V* = 350 m/min *t* = 0.1 mm *w* = 2 mm, (**c**) *V* = 100 m/min *t* = 0.2 mm *w* = 2 mm, (**d**) *V* = 100 m/min *t* = 0.05 mm *w* = 2 mm.

**Figure 4:**
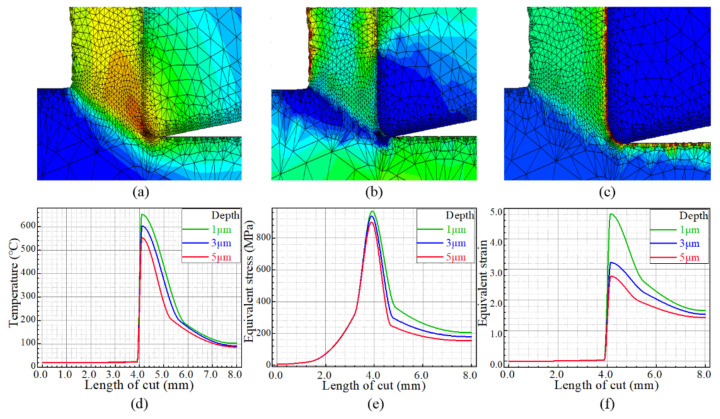
Temperature, strain and stress fields. (**a**) Temperature cloud map, (**b**) stress cloud map, (**c**) strain cloud map, (**d**) subsurface temperature variation, (**e**) subsurface stress variation, (**f**) subsurface strain variation.

According to the FE analysis, the cutting-force data could be extracted, filtered and polynomially fitted, as shown in Figure 3. Further, the temperature, stress and strain fields below the machined surface could be obtained, as shown in Figure 4.

### 2.3. Calculation Procedure

**Figure 5:**
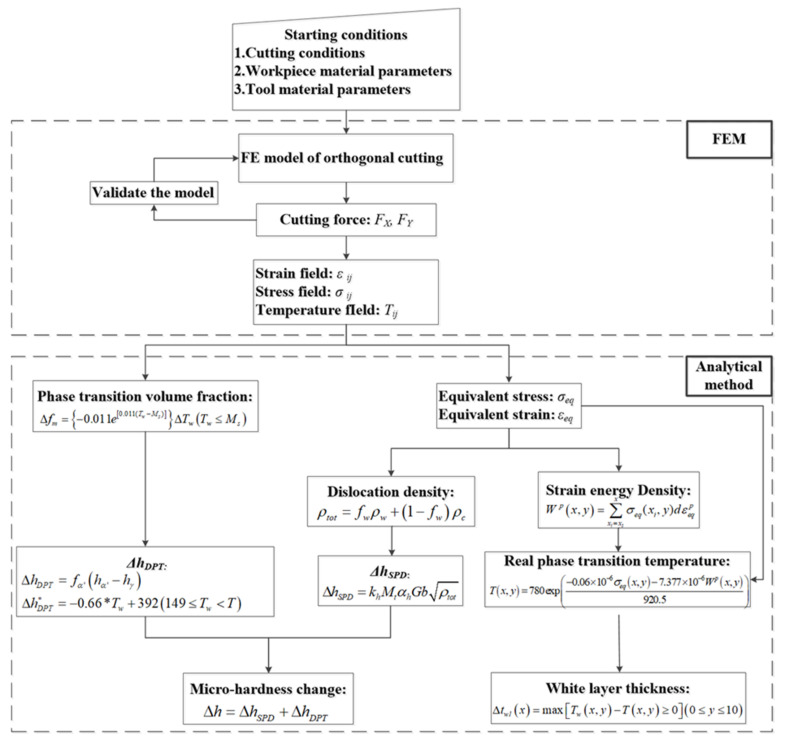
The flow chart of the methodology for the multiphysics prediction model.

As shown in the flow chart in Figure 5, a multiphysics model combining FEM and analytical method is built up for predicting microstructure changes and microhardness of AerMet100 steel. The starting condition includes cutting conditions, workpiece material properties and tool material parameters. Firstly, a FE model of orthogonal cutting is established and validated by measuring the cutting force. Then, the multiphysics fields relating to temperature, stress and strain are obtained. Subsequently, the equivalent stress and strain, dislocation density, austenite real transformation temperature, and phase-transition volume fraction are evaluated. Further, the white-layer thickness is investigated according to a function related to cutting temperature and real phase-transformation temperature. The microhardness is estimated by phase transition and dislocation density. 

## 3. Materials and Methods

### 3.1. Material and Machining Process

**Figure 6:**
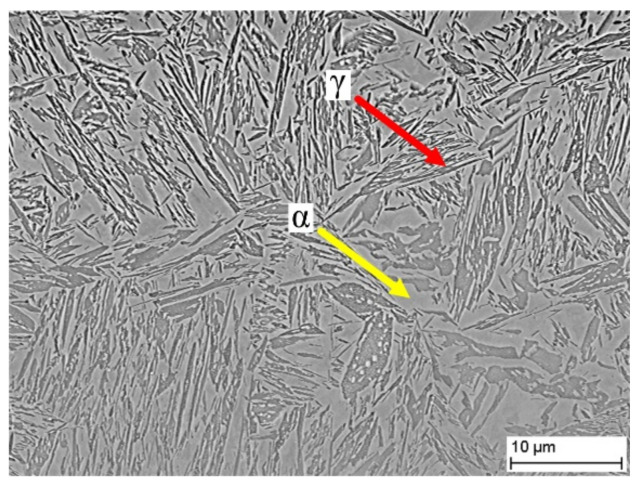
Initial microstructure of AerMet100 steel.

The workpiece material used in this study is AerMet100 steel. The main chemical compositions (wt.%) and material properties of AerMet100 steel are shown in Table 1 [29] and Table 2 [30], respectively. The metallographic morphology of AerMet100 steel is shown in Figure 6. Both acicular martensite and austenite exist together.

### 3.2. Microstructure Examination

To observe the microstructure changes after machining, metallographic samples were prepared. The specimens with the size of about 15 mm × 8 mm × 2 mm were cut from the workpiece through wire electrical-discharge machining (WEDM) and embedded in cold-mounting epoxy resin. Subsequently, all specimens were mechanically ground and polished to a mirror finish and etched using a mixture of 4% nitric acid and alcohol solution for about 5 s. After the above processes, the microstructures of the specimens were investigated using a laser-scanning confocal microscope (VK-X200K, KEYENCE, Osaka, Japan). The white-layer thickness for each specimen was determined by measuring five values at different positions. Then, the average value and standard deviation were obtained.

### 3.3. Microhardness Measurement

The microhardness measurements were performed on an automatic Vickers hardness tester (Wolpert Wilson Instruments TM, Wolpert, Shanghai, China). A test force of 25 gf was used with a holding time of 5 s. For each specimen, the microhardness measurements included two parts. Firstly, the microhardness of the machined surface was tested. Then, the subsurface microhardness profile was examined in the cross section at various depths ranging from 5 to 200 microns. It should be noted that the distance between any two measured points was at least five times the Vickers diagonal length to avoid the mutual influence among the tested points.

### 3.4. XRD Measurement

Phase analysis was carried out for both specimen surface and subsurface with X-ray diffraction (XRD-6100, Shimadzu, Kyoto, Japan). The X-ray diffractometer uses Cu Kα radiation in the glancing angle range of 30° to 95° with 8 deg/min at 40 kV and 40 mA. Moreover, for the XRD measurements in the subsurface, the specimens were electropolished 20 microns below the surface at 25 V in a mixed solution of 10% perchloric acid and 90% ethanol at 25 °C.

The orthogonal machining with a radial feed of the grooving tool was carried out by a CNC lathe with a Fanuc system. The groove cutter (TCMT 16 T3 12-UR 4325, Sandvik, Shanghai, China) with a 0 deg rake angle and a 7 deg clearance angle was operated in this study. The tool edge radius is 50 µm. Each tool was used only once. During the cutting processes, the cutting forces including tangential force (F_X_) and feed force (F_Y_) were measured with a dynamometer (9129AA, Kistler, Beijing, China). Nine cases were conducted according to the processing parameters listed in Table 3.

## 4. Results

A computer program in Matlab 2018a is developed to simulate the proposed model. The Johnson–Cook constants of AerMet100 steel are presented in Table 4 [30]. The dislocation-evolution-rate control parameters $\alpha^{*},\beta^{*}$ and $k_{0}$ are determined by the stress–strain relation [31] and set as 0.8, 0.28 and 2.9, respectively. The dislocation-density-based model parameters, listed in Table 5, are referred to the reference [23].

The model validation includes four parts: (1) evaluation of cutting force, (2) evaluation of phase transformation, (3) evaluation of white-layer thickness and (4) evaluation of microhardness. The input-cutting parameters are corresponding to the experimental conditions listed in Table 3.

### 4.1. Cutting Force

**Figure 7:**
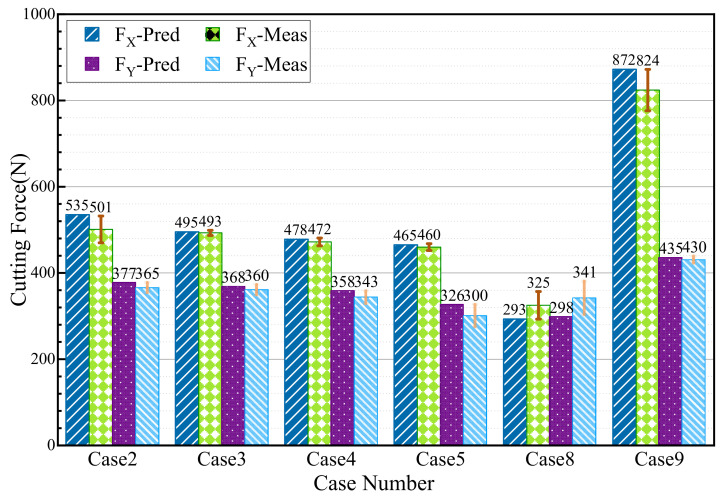
Comparison of predicted cutting forces with the experimental results.

The measured and evaluated cutting forces are compared in Figure 7. Take Case 2 for instance: the predicted cutting forces are 535 N and 377 N in the cutting direction and the perpendicular direction, while the measured results are 501 N and 365 N, respectively. The largest difference between the predicted and experimental results is about 13% in the perpendicular direction of Case 8. From the comparison, the predicted cutting forces are consistent with the experimental data, indicating the effectiveness of the FE model for orthogonal cutting. The FE cutting model provides a foundation for evaluating the multiphysics fields of the machined subsurface.

### 4.2. Phase Transformation

**Figure 8:**
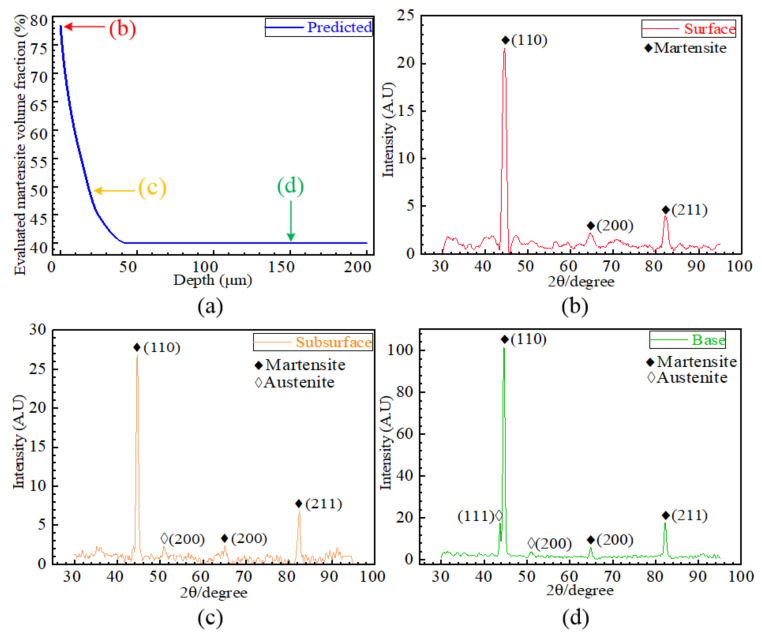
The evaluated martensite volume fraction and XRD analysis for phase change. (**a**) The evaluated martensite fraction, (**b**) surface diffractogram, (**c**) subsurface diffractogram, (**d**) base diffractogram.

The evaluation of martensite volume fraction along the subsurface of Case 4 is shown in Figure 8a. The evaluated martensite volume fraction is about 79% on the surface and drastically decreases with the increasing depth. Other cases present a similar variation of martensite volume fraction. Figure 8b–d show the results of XRD analysis, which were conducted on the surface, subsurface of 20 μm deep and the base material, respectively. Only martensite existed on the surface, as revealed by Figure 8b. Moreover, from the XRD analysis in Figure 8c, martensite peaks as well as a typical austenite peak are observed, suggesting a lower martensite volume fraction at the depth of 20 μm. Further, compared the XRD results in Figure 8c,d, the austenite peaks are more obvious in the base material, which suggests a higher austenite volume fraction in the base material than that in the subsurface of about 20 μm deep. From the comparison, the evaluated results of martensite volume fraction are in good agreement with the XRD analysis.

### 4.3. White-Layer thickness

**Figure 9:**
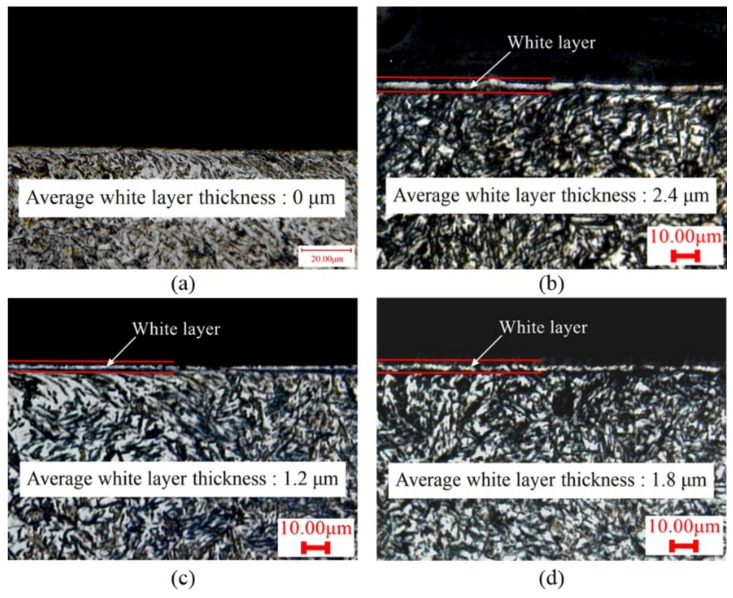
The microstructure of AerMet100 steel generated in experiments: (**a**) Case 1, (**b**) Case 4, (**c**) Case 8, (**d**) Case 9.

**Figure 10:**
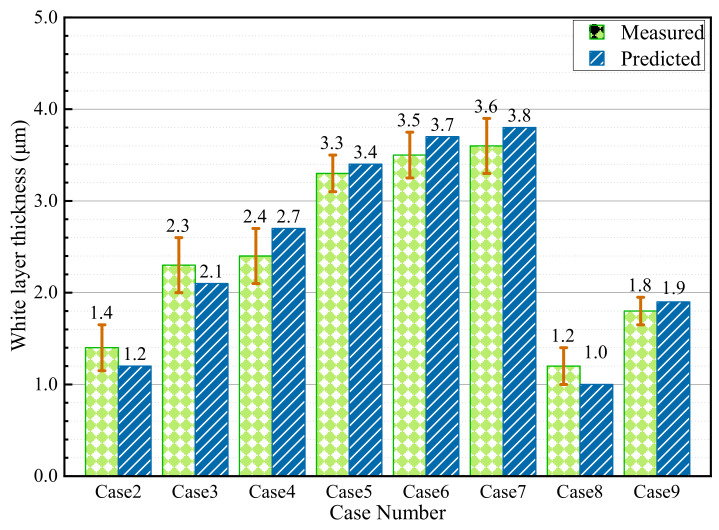
White-layer thickness of AerMet100 steel.

The microstructure morphologies of Cases 1, 4, 8 and 9 are shown in Figure 9. No white layer was observed on the machined surface of Case 1, while the white layers are obvious in other cases. In addition, from the subsurface metallographic structure as shown in Figure 9, the grains are broken and distorted near the top surface, indicating serious plastic deformation induced by machining. Furthermore, the white-layer thickness was measured and compared with the predicted result, as displayed in Figure 10. Take Case 5 for instance: the evaluated and measured white-layer thicknesses are 3.4 µm and 3.3 µm, respectively. The vastest difference between the evaluated and measured results is 17% in Case 8. The predicted results are in good agreement with the measured results.

### 4.4. Microhardness

In this study, the measured microhardness is based on Vickers hardness HV while the predicted microhardness of the model is based on GPa. For the sake of comparison, the two microhardness units are unified by the formula 1 GPa = 102 HV [32]. The microhardness of the AerMet100 steel matrix is 370 HV (3.63 GPa) [30]. The microhardness of martensite and austenite are 571 HV (5.6 GPa) and 190 HV (1.863 GPa), respectively [23].

**Figure 11:**
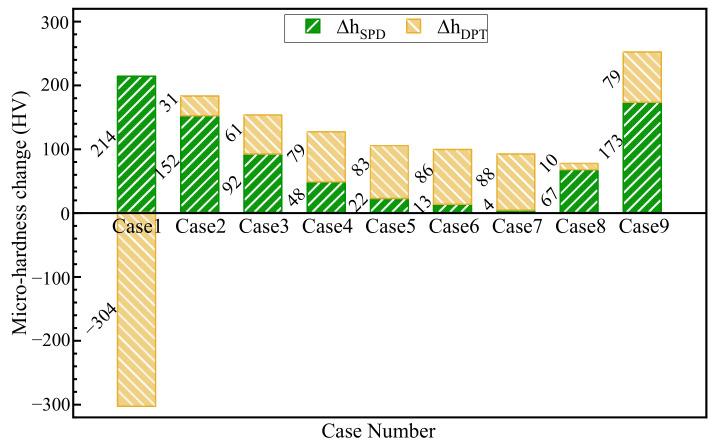
Predicted values of surface microhardness change.

**Figure 12:**
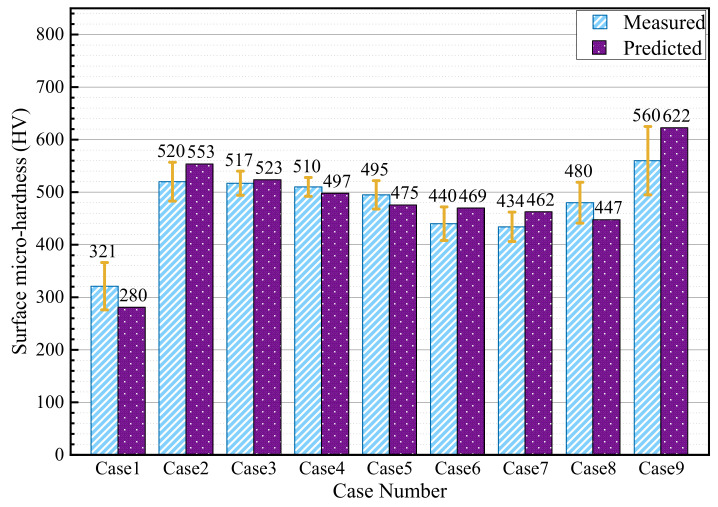
Predicted and measured surface microhardness.

For each case, the microhardness changes resulted from plastic deformation and phase transformation are respectively evaluated, as displayed in Figure 11. For instance, the microhardness changes due to plastic deformation and phase transformation are 214 HV and −304 HV in Case 1 while those are 31 HV and 152 HV in Case 2. Moreover, according to the evaluated microhardness changes, the resultant microhardness of each surface is obtained and compared with the measured result, as shown in Figure 12. Take Case 3 as an example: the predicted and measured surface microhardnesses are 523 HV and 517 HV, respectively. The largest difference between the predicted and experimental results is about 13% in Case 1.

**Figure 13:**
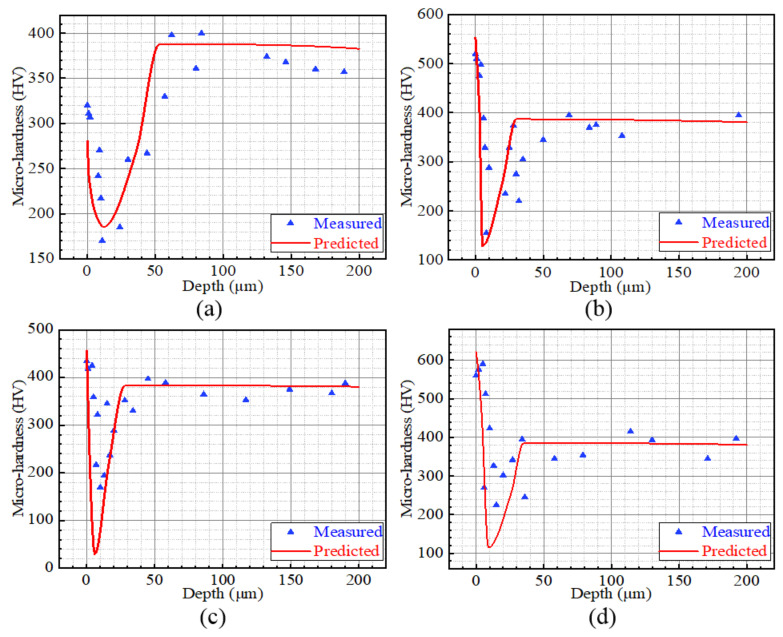
Evaluated and measured microhardness profiles: (**a**) Case 1, (**b**) Case 2, (**c**) Case 7, (**d**) Case 9.

In terms of the microhardness along the depth, the predicted and measured result distributions of Cases 1, 2, 7 and 9 are presented in Figure 13. It is clear that the variation of predicted values is consistent with the measured results to a certain degree. The microhardness profile presents a spoon-shaped variation, which is consistent with the study reported by Umbrello et al. [33]. The microhardness firstly deceased and then increased with the increasing depth. It is noted that both the deformation of the local material and the local microstructure are not uniform, so the measured microhardness has a certain discreteness.

## 5. Discussion

According to the predicted and measured results, different process parameters result in different white layer depth and microhardness changes. The correlation between processing parameters and surface characteristics is further investigated.

### 5.1. White Layer

**Figure 14:**
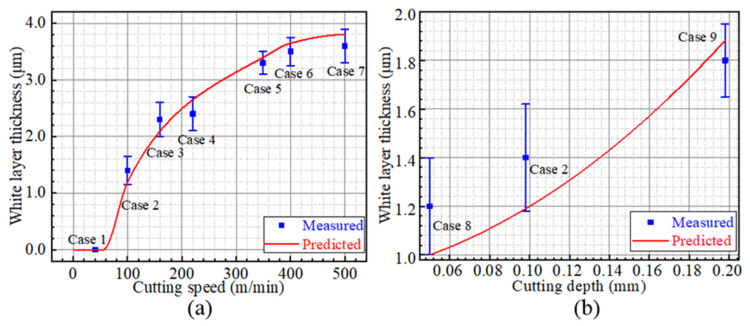
White-layer thickness prediction curves: (**a**) The relationship between white-layer thickness and cutting speed, (**b**) The relationship between white-layer thickness and cutting depth.

**Figure 15:**
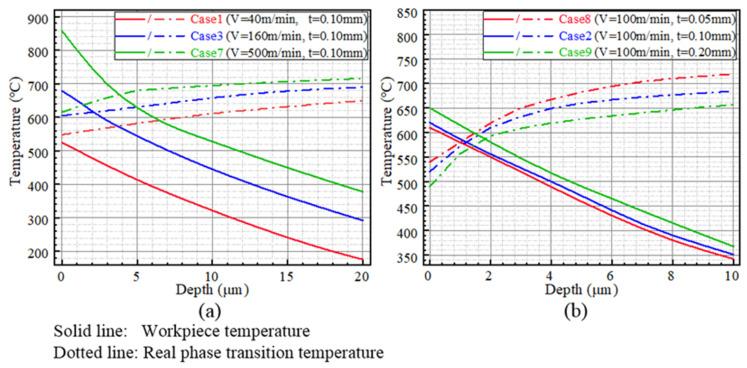
Evaluation of white-layer thickness: (**a**) The relationship between temperature and cutting speed, (**b**) The relationship between temperature and cutting depth.

The relationship between white-layer thickness and cutting speed is depicted in Figure 14a. The white-layer thickness increases with the increase in cutting speed. This can be attributed to the plastic deformation and workpiece temperature increase induced by machining. Based on the proposed model, the evaluated workpiece-temperature profiles and real phase-transition temperature variations of Cases 1, 3 and 7, which suffered the same cutting depth of 0.10 mm and the increasing cutting speeds of 40, 160 and 500 m/min, are investigated. With the increase in cutting speed, the cutting force reduces [34], and more cutting heat is generated [21]. Due to the reduced cutting force and increased workpiece temperature, both the real phase-transition temperature and workpiece temperature increase with the increase in cutting speed, as shown in Figure 15a. Consequently, the austenite structures were produced at the deeper position of the subsurface due to the increased cutting speed, which leads to the increase in white-layer thickness. Another attractive phenomenon is that there exists a threshold of cutting speed for white-layer generation. No white layer would be formed when the cutting speed is smaller than the threshold (approximately 60 m/min when the cutting depth is kept at 0.1 mm in the present study).

Furthermore, the relationship between cutting depth and white-layer thickness is shown in Figure 14b. The white-layer thickness increases with the increase in cutting depth. This also can be attributed to the stress and temperature, which increase significantly with the increasing cutting depth [22]. Both the stress induced by the plastic deformation and the heat that transfers into the subsurface provide the driving force for white-layer formation. As shown in Figure 15b, on the one hand, the real phase-transition temperature is decreased as the result of the increasing stress. The austenite phase transformation is significantly promoted along the subsurface. On the other hand, as a result of increased temperature, austenitic transformation occurs at a deeper position of the subsurface. Consequently, the white-layer thickness shows an upward trend with the increasing cutting depth.

### 5.2. Microhardness

**Figure 16:**
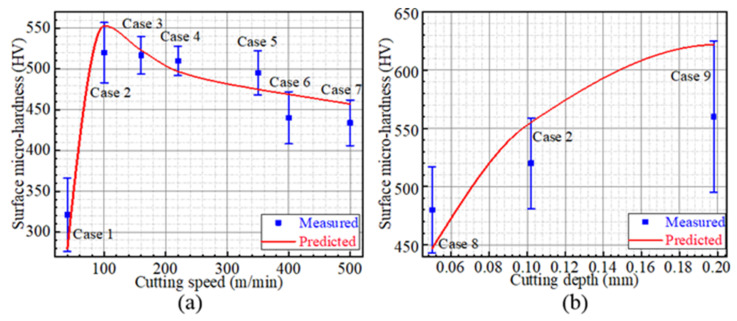
The surface microhardness curves: (**a**) The relationship between micro-hardness and cutting speed, (**b**) The relationship between micro-hardness and cutting depth.

The relationship between cutting speed and surface microhardness is displayed in Figure 16a. With the increase in cutting speed, the microhardness increases significantly when the speed is lower than 105 m/min and decreases slightly when the speed is higher than 105 m/min. Take Cases 1 and 2, which suffered the cutting speeds of 40 and 100 m/min, for instance: No white layer was formed in Case 1, while the white layer was produced in Case 2. It indicates that no austenite transformation occurs on the machined surface of Case 1 and the machined surface is tempered. In particular, the microhardness change due to the tempering effect of Case 1 is −304 HV, as shown in Figure 11. Therefore, the microhardness has a significant change under low-cutting-speed conditions as the result of white-layer formation. Under moderate-high-speed cutting conditions, the microhardness decreases slightly with the increase in cutting speed. The microhardness changes of Cases 3 and 7, which suffered the increasing cutting speeds of 160 and 500 m/min, are compared. As displayed in Figure 11, the microhardness changes due to plastic deformation and phase transformation are 92 HV and 61 HV in Case 3, while those are 4 HV and 88 HV in Case 7. The microhardness change due to plastic deformation is markedly decreased, since the cutting force is decreased with the increasing cutting speed [28]. Furthermore, the microhardness change due to phase transformation is increased, as the increasing cutting speed leads to more cutting heat [21]. Combined with the results of Figure 11, the thermal effects predominate over mechanical effects for microhardness when the cutting speed ranges from 105 to 500 m/min.

Moreover, the relationship between microhardness and cutting depth is shown in Figure 16b. The microhardness increases with the increase in cutting depth. The microhardness changes of Cases 8, 2 and 9, which suffered the same cutting speed of 100 m/min and the increasing cutting depths of 0.05, 0.10 and 0.20 mm, are investigated. For Cases 8, 2 and 9, the microhardness changes due to plastic deformation are 67 HV, 152 HV and 173 HV, and the microhardness changes due to phase transition are 10 HV, 31 HV and 79 HV, as shown in Figure 11. The microhardness increment analysis reveals that the increasing cutting depth brings about the increase in microhardness change due to both plastic deformation and phase transition, which can be attributed to the large cutting force and cutting heat, respectively.

In addition, the microhardness profiles present a spoon-shaped variation. According to the analysis proposed by Grange et al. [25], the tempering zone, which is the area within the tempering-temperature gradients, could produce tempered martensite with low microhardness. In the present study, take Case 7, for instance: the tempering zone corresponds to the subsurface zone with the depth range of 3.8–26.5 μm and suffered temperatures of about 149–668 °C as estimated by the proposed model. Within this zone, a considerable amount of tempered martensite with low microhardness is produced as illustrated by the XRD analysis and the microhardness results.

In summary, the white-layer thickness and surface microhardness are highly related to the cutting parameters. The relationship between white-layer thickness, surface microhardness and cutting parameters can be summarized as follows. Under low-speed cutting conditions, the machined surface had lower microhardness and no white layer was formed. When the cutting speed reached a value of about 105 m/min, the white layer was produced and the microhardness was the greatest on the surface. Under moderate-high-speed cutting conditions, the white-layer thickness increased, while the microhardness decreased with the increase in cutting speed. Both white-layer thickness and microhardness increased with the increase in cutting depth. According to the previous analysis, the cutting parameters relate the plastic deformation and temperature of the workpiece surface by affecting the cutting force and cutting heat, which finally result in the microhardness change and white-layer generation. To reveal the quantitative correlation between the machining parameters and surface/subsurface characteristics, a multiphysics model is established in the present study. Importantly, the correlation suggests that the white-layer thickness and the surface microhardness can be regulated by processing parameters.

## 6. Conclusions

A multiphysics model is proposed to predict the machining-induced microstructure changes and microhardness in orthogonal cutting of AerMet100 steel. The accuracy and effectiveness of the proposed model are validated with experimental results. The proposed model is proven to be a comprehensive way for assessing crucial microstructure attributes induced by machining. The main conclusions are as follows:

1. A prediction model for white-layer thickness and microhardness is established, and the machining-induced phase transformation, white-layer generation and microhardness change can be evaluated through the variations of stress, strain and temperature. The predicted results are in good agreement with the experimental data.
2. White-layer thickness is evaluated considering phase transformation and stress/strain state. There is a remarkable influence of cutting speed on the white-layer thickness since the workpiece temperature rises significantly with the increasing cutting speed.
3. The microhardness change is mainly related to the dislocation density and phase transformation. The surface microhardness could be softened or hardened under different cutting conditions. The microhardness profile presents a spoon-shaped variation.
4. The white-layer formation and microhardness change are highly related to cutting conditions. The present study provides a theoretical basis for controlling surface microstructure and microhardness by selecting processing parameters for industrial applications.

## Figures and Tables

**Table 1:** Chemical compositions of AerMet100 steel [29].

C	Mn	Si	Ni	Cr	Mo	Al	Co	Ti	O	N	S+P	Fe
0.225	0.01	0.01	11.22	3.04	1.20	0.015	13.50	≤0.015	≤0.002	≤0.0015	0.01	balance

**Table 2:** Material properties of AerMet100 steel [30].

Thermal Conductivity (W/m·°C)	Thermal Diffusivity (m^2^/s)	Elastic Modulus (GPa)	Poisson’s Ratio	Yield Strength (MPa)	*T_m_* (°C)	*T_r_* (°C)	Density (kg/m^3^)	Specific Heat (J/kg·°C)
19.3	5.9 × 10^−6^	206	0.3	831.8	1460	20	7889	412.7

**Table 3:** Machining parameters for orthogonal cutting.

Case	Cutting Speed (m/min)	Cutting Depth (mm)	Cutting Width (mm)
1	40	0.10	2
2	100	0.10	2
3	160	0.10	2
4	220	0.10	2
5	350	0.10	2
6	400	0.10	2
7	500	0.10	2
8	100	0.05	2
9	100	0.20	2

**Table 4:** Johnson–Cook constants for orthogonal machining of AerMet100 steel [30].

A (MPa)	B (MPa)	C	m	n
831.8	731.3	0.01	0.8571	0.2893

**Table 5:** Dislocation-density-model parameters for AerMet100 steel [23].

$n_{0}$	${\dot{\gamma }}_{0}$	$f_{0}$	$f_{\infty }$	$K_{0}$	${\tilde{\gamma }}^{r}$	*G* (GPa)	$M_{t}$	$\rho_{c}^{0}\;(m^{-2})$	$\rho_{w}^{0}\;(m^{-2})$	b (m)
50	10^3^	0.25	0.07	10	2.5	80	3.06	1.3 × 10^10^	1.21 × 10^11^	2.48 × 10^−10^

## Data Availability

Not applicable.

## Nomenclature

V,t,m	speed, depth, width of cut
$\bar{\sigma },\sigma_{eq}$	equivalent stress
$\bar{\varepsilon },\varepsilon_{eq}$	equivalent strain
A,B,C,m,n	material parameters of Johnson–Cook model
μ	friction coefficient
$F_{X},F_{Y}$	tangential force and feed force
$T_{w},T_{m},T_{r}$	workpiece temperature, melting temperature and room temperature
$T_{0}$	nominal phase-transition temperature
T	real phase-transition temperature
$T_{ij},\sigma_{ij},\varepsilon_{ij}$	temperature, stress and strain fields (*i, j*=*x, y*)
$\sigma_{x},\sigma_{y},\sigma_{z},\sigma_{xy}$	stress component
$\Delta_{\alpha^{\prime }}^{\gamma }V,\Delta_{\alpha^{\prime }}^{\gamma }H$	molar volume increment and molar latent heat
W	strain energy density
$d\varepsilon_{eq}^{p}$	equivalent plastic strain increment
h	microhardness
$\Delta h_{SPD}$	microhardness change due to severe plastic deformation
$\Delta h_{DPT}$	microhardness change due to dynamic phase transformation
$\Delta h_{DPT}^{*}$	microhardness change due to the tempering effect
b	the magnitude of the Burgers vector of the material
$\rho_{tot}$	total dislocation density
$\rho_{c},\rho_{w}$	dislocation densities of cell interior and walls
${\dot{\gamma }}_{c},{\dot{\gamma }}_{w}$	resolved shear-strain rates for cell interiors and walls
${\tilde{\gamma }}^{r}$	the reference resolved shear strain
${\dot{\gamma }}_{0}$	the reference resolved shear-strain rate
$\gamma^{r}$	resolved shear strain
${\dot{\gamma }}^{r}$	resolved shear-strain rate
d	average cell size
$f_{w}$	volume fraction of the dislocation cell wall
$f_{0},f_{\infty }$	initial and saturation volume fractions of cell walls
f	Phase-volume fraction
